# Adding Person-Centered Measures to Research on Detection and Diagnosis of Dementia

**DOI:** 10.1093/geroni/igab046.1009

**Published:** 2021-12-17

**Authors:** Katie Maslow

**Affiliations:** Gerontological Society of America, Washington, District of Columbia, United States

## Abstract

In the United States, numerous studies on detection and diagnosis of dementia show that large proportions of subjects refuse initial screening tests. Moreover, among those who accept the tests, score poorly, and are therefore referred for a diagnostic evaluation, large proportions do not follow up to get the evaluation. Available data on characteristics of subjects who refuse initial screening and follow-up evaluation suggest that incorporating procedures based on person-centered concepts and practices, such as procedures that acknowledge individuals’ unique characteristics and attempt to involve, enable, and empower them, could lead to more effective detection and diagnosis. Based on results of an analysis of measures used in studies conducted in the U.S. and elsewhere, this presentation will describe frequently used measures and identify person-centered measures that could be added to studies of alternate procedures intended to increase detection and diagnosis.

